# Development and Application of a Pseudovirus-Based Assay for Modelling SARS-CoV-2 Spike Protein Mediated Drug Screening

**DOI:** 10.3390/ijms27020791

**Published:** 2026-01-13

**Authors:** Shokhrukh A. Khasanov, Iana L. Esaulkova, Alexandrina S. Volobueva, Alexander V. Slita, Daria V. Kriger, Dmitri Tentler, Olga I. Yarovaya, Anastasia S. Sokolova, Andrey N. Gorshkov, Anna S. Dolgova, Irina N. Lavrentieva, Vladimir G. Dedkov, Nariman F. Salakhutdinov, Vladimir V. Zarubaev

**Affiliations:** 1 Saint Petersburg Pasteur Institute, Federal Service for the Oversight of Consumer Protection and Welfare, 197101 Saint Petersburg, Russia; hasanov@pasteurorg.ru (S.A.K.);; 2 Division of Pediatric Infectious Diseases, Rambam Health Care Campus, Haifa 3109601, Israel; 3 Institute of Cytology, Russian Academy of Sciences, 194064 Saint Petersburg, Russia; 4 G-INCPM, the Weizmann Institute of Science, Rehovot 7610001, Israel; 5 N. N. Vorozhtsov Novosibirsk Institute of Organic Chemistry, 630090 Novosibirsk, Russia; 6 A. A. Smorodintsev Influenza Research Institute, 197376 Saint Petersburg, Russia; 7 Martsinovsky Institute of Medical Parasitology, Tropical and Vector Borne Diseases, Sechenov First Moscow State Medical University, 119435 Moscow, Russia

**Keywords:** coronavirus, SARS-CoV-2, ACE2, spike protein, lentivirus, pseudotyping, H1299, antivirals

## Abstract

Requirements for novel effective antiviral agents against SARS-CoV-2 emphasizes the importance of robust in vitro screening platforms. We developed a test system based on spike-pseudotyped lentiviruses, carrying either *luc+* or EGFP reporter genes as a payload, and a human non-small cell lung carcinoma (NSCLC) cell line, overexpressing ACE2 (H1299-hACE2). The cell origin makes our system resemble lung epithelium infection. Transmission electron microscopy confirmed that the spike glycoproteins on the pseudotyped lentiviral particles resemble native SARS-CoV-2 spike glycoproteins, thus validating their use in inhibitor screening. H1299-hACE2 cells showed significantly higher infection rate (*p* < 0.005) with spike-pseudotyped lentiviruses compared to parental H1299 cells, as determined by luciferase and fluorescence assays. The susceptibility of the stable H1299-hACE2 cell line to a broad panel of SARS-CoV-2 variants (Wuhan, Beta, Delta, Omicron) was assessed here for the first time in a unified experimental setting. Infection of H1299-hACE2 cells with SARS-CoV-2 induced cell fusion and syncytium formation with subsequent cell death. The developed pseudovirus-based assay was further used for assessment of the antiviral properties of derivatives of 1,7,7-trimethyl-[2.2.1]-bicycloheptane-potential spike protein inhibitors, which possess moderate activity against lentiviral particles. The H1299-hACE2/spike-pseudotyped lentivirus assay is, therefore, a reliable, high-efficiency platform for screening spike-mediated entry inhibitors. The cell line obtained during the development of the platform can be used to isolate and study new variants of SARS-CoV-2.

## 1. Introduction

Until recently, coronaviruses have not been considered serious human pathogens. Common human coronaviruses (229E, OC43, NL63, HKU1) account for 15–20% of seasonal acute respiratory viral infections, leading mostly to mild disease. In 2003, however, a large outbreak of the severe acute respiratory syndrome coronavirus (SARS-CoV) resulted in 8422 cases including 919 deaths [1]. In 2013, an outbreak of Middle East respiratory syndrome (MERS) led to 2494 cases and 858 deaths across 27 countries [2]. Finally, the emergence of SARS-CoV-2 resulted in the COVID-19 pandemic, which spread throughout the world. As of June 2025, official estimates indicate 777,825,189 registered cases and over 7 million deaths [3].

Coronavirus particles are enveloped virions, 80 to 90 nm in diameter [4], containing non-segmented, single-stranded RNA of positive polarity (26,000 to 37,000 bases in length). The virion consists of four structural proteins: the spike protein (S), which is responsible for attachment to the host cell surface and subsequent membrane fusion; the nucleocapsid protein (N), which packages the viral genome; and two envelope-associated proteins—the membrane protein (M) and the envelope protein (E) [5]. The S protein binds to cellular receptor, angiotensin converting enzyme 2 (ACE2), and mediates fusion of viral envelope with the host plasma membrane, leading to the release of the viral genome into the cytoplasm [6]. As the positive-sense RNA genome is capped and polyadenylated, it can function directly as mRNA for translation by host ribosomes. Upon entry, the viral RNA translates and undergoes proteolytic processing, resulting in the synthesis of 4 structural and 14 non-structural proteins (*nsp*). The latter are responsible for suppressing innate immunity and facilitating efficient genome replication. The viral polymerase (transcriptase–replicase) complex composed of non-structural proteins including *nsp7*, *nsp8* and *nsp12* directs the synthesis of a complementary negative-strand RNA, which serves as a template for producing new positive-strand genomic RNA [7]. Subsequently, the newly synthesized genomic RNA, together with newly produced structural proteins, assembles into progeny virions that bud into the lumen of the Golgi apparatus. These virions are then transported via vesicles and released into the extracellular space. [8].

Various members of the *Coronaviridae* family can infect humans and other mammals, such as camels, bats, civets, cats and pigs [9]. Consequently, the viral entry protein (spike protein) can bind to different host cell surface receptors on the surface of the host cell, depending on the specific coronavirus and its host. For example, α-coronaviruses primarily use aminopeptidase N as a receptor [10], while β-coronaviruses use dipeptidyl peptidase 4 (DPP4) [11,12] or angiotensin-converting enzyme 2 (ACE2) [13,14]. All receptors used by coronaviruses for cell entry are essential for mammalian cells and are therefore widely distributed in their organisms. All known human coronaviruses were transmitted to the human population from animals (directly or through an intermediate host): SARS-CoV-2, SARS-CoV, MERS-CoV, HCoV-NL63 and HCoV-229E–from bats; HCoV-OC43 and HKU1 from rodents [15].

The zoonotic origin of SARS-CoV-2 is supported by genomic and epidemiological evidence indicating its transmission from animal reservoirs to humans [16,17]. Bats are considered the primary reservoir, with potential intermediate hosts such as pangolins or raccoon dogs enabling recombination events. These events introduced key spike protein features, including optimizations in the receptor-binding domain and the acquisition of a furin cleavage site, which enhanced human transmissibility [18,19,20]. The S protein’s role in this zoonotic jump is central, as it mediates viral entry primarily by binding ACE2 to host cells. This receptor is conserved across many mammals, but the SARS-CoV-2 spike protein has evolved with human-specific affinities [21,22]. Successful entry is further facilitated by additional host factors including co-receptors and the transmembrane serine protease 2 (TMPRSS2), which primes the S protein for membrane fusion [6].

Several vaccine types have been developed and utilized in order to control the dissemination of SARS-CoV-2 infection among the human population. These vaccines differ in efficacy, immunogenicity and safety profile, and are based on various technological approaches, such as inactivated whole-virion vaccines, RNA-based or adenoviral vector-based vaccines [23,24,25]. Low-molecular-weight compounds of various origin (novel, re-purposed) and mechanism (direct-acting, host component targeting) have also been used to treat COVID-19 treatment with varying degrees of success [26,27]. Despite these advances in vaccine and antiviral drug development, the infection is far from being eliminated, or even robustly controlled. Furthermore, due to the high virulence and pathogenicity of SARS-CoV-2, all work with the infectious virus must be carried out under stringent biosafety conditions. To enable safer study of viral entry mechanisms without handling the live virus, a pseudotyping methodology has been developed.

The possibility of creating recombinant lentiviruses was first investigated in 1989 [28]. In general, virus pseudotyping is the process of replacing the original surface glycoprotein of an enveloped virus with a surface glycoprotein from a different enveloped virus. Lentiviruses, like other retroviruses, can incorporate glycoproteins from other viruses into their envelope membrane. This technology was first demonstrated for replication-defective lentiviruses using the amphotropic envelope of Moloney murine leukemia virus 4070A (A-MoMLV) [29] and the envelope of human T-cell leukemia virus type I (HTLV-I) [30]. The vesicular stomatitis virus glycoprotein (VSV-G) is one of the most used envelope proteins. This preference is due to its broad cellular and tissue tropism, high stability, and the stability it confers to lentiviral vectors, which makes concentration by ultracentrifugation possible [31,32].

The pseudotyping technology for enveloped viruses, such as lentiviruses and vesicular stomatitis virus (VSV), is now widely used to study highly pathogenic viruses that normally require the highest (BSL-4) biosafety containment. For instance, modelling the envelopes of filoviruses like Marburg virus (MARV) and Ebola virus (EBOV), which are the most lethal viruses, can be performed in standard BSL-2 biosafety laboratories. This approach enables the safe study of viral entry mechanisms, screening for entry inhibitors, detection of neutralizing antibodies, and analysis of glycoprotein mutations [33].

The emergence of SARS-CoV-2 in late 2019 created an urgent need for safe and effective tools to search for virus entry inhibitors without the need to work in biosafety level 3 (BSL-3) laboratories. Pseudotyping technology, which involves incorporating the SARS-CoV-2 spike (S) protein into the envelope of non-pathogenic viruses such as VSV and lentiviruses, has enabled research to be conducted under BSL-2 conditions. Pseudotyped systems developed in 2020 allowed for the rapid study of viral entry mechanisms, interaction with the host receptor ACE2 and the protease TMPRSS2, and the quantitative assessment of neutralizing antibody activity using reporter genes [34,35,36]. Significant improvements were subsequently introduced to the spike-pseudotyped lentivirus systems, such as truncating the C-terminal region of the S protein (e.g., 19 amino acids), which increased protein incorporation and pseudovirus titres, thereby enabling high-throughput screening [36,37].

In this study, we aimed to develop a pseudovirus-based system for screening SARS-CoV-2 entry inhibitors. Our goal was to combine the advantages of existing assays, such as flexible detection methods, the use of a lung-derived cell line, stable *ACE2* expression and high infection rate. We subsequently established a robust pseudovirus assay using a luciferase reporter gene and employed it to evaluate a series of bicyclic monoterpenoid and labdane-type diterpenoid derivatives for their inhibitory activity against SARS-CoV-2 spike protein-mediated entry.

## 2. Results

### 2.1. Developing the Pseudovirus-Based System for Screening of Potential Spike–ACE2 Interaction and Fusion Inhibitors

In this study, we aimed to develop an assay for assessing spike protein activity that would (i) be suitable for evaluating inhibitor efficacy and (ii) resemble, as closely as possible, natural coronavirus infection in terms of target cell histological lineage. To this end, we employed a highly effective lentiviral system commonly used for gene delivery into human cells. As described in the Materials and Methods section, the system utilizes three plasmids encoding the following: (1) the VSV-G protein, which mediates viral entry into cells; (2) the proteins required for viral RNA synthesis and packing; and (3) the payload RNA. To confer specificity for SARS-CoV-2, the gene encoding VSV-G was replaced with a codon-optimized *spike* gene. This modification was designed to achieve a high level of spike protein expression, sufficient for effective assembly of pseudoviral particles. To ensure sensitive detection of infected cells, we constructed two delivery plasmids with *luc+* or *EGFP* genes. This offers flexibility in using various detection systems, depending on the experimental needs.

The H1299 cell line was chosen due to its origin from lung epithelium, which closely resembles the natural SARS-CoV-2 target cells. To increase infection rates and make the system ACE2-specific, we generated an H1299 cell line with stable overexpression of the *ACE2* gene. In result, we constructed a system for the evaluation of spike-ACE2 mediated infection. The system consists of spike-pseudotyped lentiviruses carrying a *luc+* reporter gene and a lung cancer cell line designed for highly efficient SARS-CoV-2 infection. To control for non-specific effects and specifically screen for inhibitors of spike-ACE2 interaction and membrane fusion, the system also includes parallel assays using VSV-G-pseudotyped lentiviruses.

### 2.2. Visualisation of Spike/VSV-G-Pseudotyped Lentiviruses

**Figure 1:**
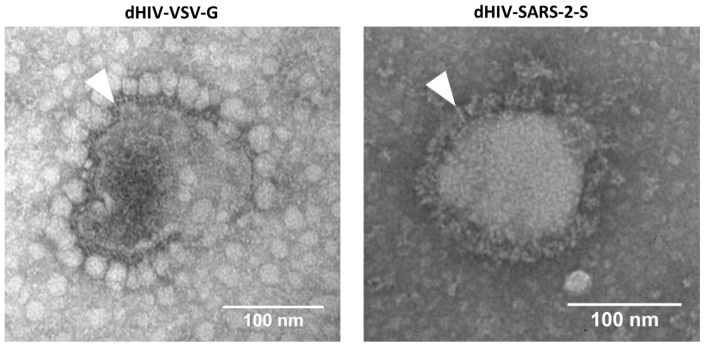
TEM images of control lentivirus (VSV-G-pseudotyped, (**left**)) and SARS-CoV-2 spike-pseudotyped lentivirus (**right**). Surface fusion proteins are indicated by arrowheads.

The spike and VSV-G-pseudotyped lentiviruses were produced, and their surface glycoproteins were characterized using transmission electron microscopy (Figure 1). Visual analysis showed that the spike glycoproteins on the lentiviral particles closely resembled those of the SARS-CoV-2 virus glycoproteins documented in previous studies [38,39]. This structural similarity validates the use of these pseudovirions for screening viral entry inhibitors. Visual analysis showed that VSV-G on the surface of the control pseudotyped lentivirus were similar in size to the glycoproteins of the VSV-G-pseudotyped lentivirus studied previously [40].

### 2.3. The Stable H1299-hACE2 Cell Line Is Highly Permissive to SARS-CoV-2 Virus and Spike-Pseudotyped Lentiviruses

**Figure 2:**
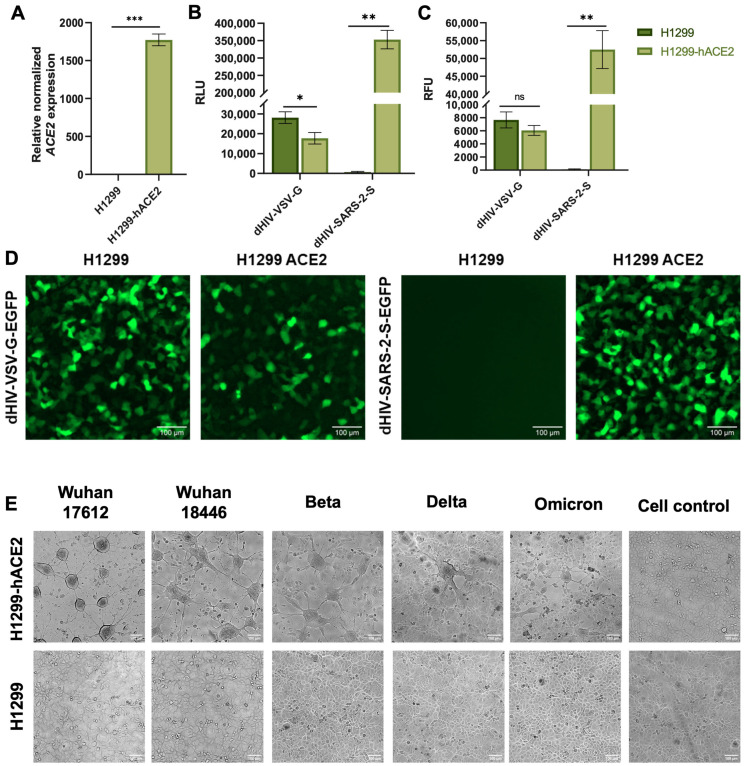
(**A**) Relative expression of ACE2 in H1299 and H1299-hACE2 cell lines. (**B**) Measurement of luciferase activity in H1299 and H1299-hACE2 cells (*n* = 6) transfected with dHIV-VSV-G-Luc and dHIV-SARS-2-S-Luc pseudoviruses. (**C**) Fluorescent analysis of H1299 and H1299-hACE2 cells (*n* = 6) transfected with dHIV-VSV-G-EGFP and dHIV-SARS-2-S-EGFP pseudoviruses. Statistical significance: *p* < 0.05 (*), *p* < 0.01 (**), *p* < 0.001 (***); ns, not significant. (**D**) Transduction of H1299 and H1299-hACE2 cell lines by pseudotyped lentiviruses. The dHIV-VSV-G-EGFP pseudovirus infects both cell lines similarly. The dHIV-SARS-2-S-EGFP pseudovirus effectively infects only cells with ACE2 overexpression. (**E**) H1299 and H1299-hACE2 cell lines infected with SARS-CoV-2 virus (Wuhan strain 17612, Wuhan strain 18446, Beta, Delta and Omicron). H1299-hACE2 cells fuse and form multicellular structures 24 h after infection. In contrast, H1299 cells show no cytopathic effects. Cell control—non-infected cells.

Initially, the H1299-hACE2 cells were established and verified *ACE2* gene expression levels by qRT-PCR. The results confirmed that *ACE2* mRNA levels in H1299-hACE2 cells were significantly higher than in the original H1299 cell line (*p* < 0.001, Student’s *t*-test) (Figure 2A). For raw data see Supplementary Tables S1 and S2. Next, we assessed the susceptibility of both cell lines, H1299 and H1299-hACE2, to infection by SARS-CoV-2 spike-pseudotyped lentiviruses (Figure 2D). Luciferase and fluorescence assays demonstrated that H1299-hACE2 cells were infected significantly more efficiently by spike-pseudotyped lentiviruses than the original H1299 cells (*p* < 0.005 in both cases, Mann–Whitney U test) (Figure 2B,C). In contrast, control (VSV-G-pseudotyped) lentiviruses infected both cell lines with similar efficiency. For raw data see Supplementary Tables S3 and S4

To study virus-induced cytopathic effects, we infected H1299-hACE2 cells and H1299 cells with several lines of authentic SARS-CoV-2: Wuhan strain 17612, Wuhan strain 18446, Beta, Delta and Omicron. Infected H1299-hACE2 cells were shown to exhibit cell fusion with syncytium formation (Figure 2E). In contrast, the original H1299 cell line exhibited no such cytopathic effects. Pseudovirus-infected cells exhibited no cytopathic effects. For raw data see Supplementary Tables S8 and S9 and Figure S1.

### 2.4. Cytotoxicity Evaluation 

The cytotoxicity data are presented in Table 1. Compounds **3**, **4**, **5**, and **6** showed no cytotoxic activity at the tested concentrations (CC_50_ > 119.8 μM, CC_50_ > 119.8 μM, CC_50_ > 107.7 μM, CC_50_ > 103.3 μM). In contrast, labdanediol **7** was highly toxic to H1299-hACE2 cells (CC_50_ value from 15 ± 0.46 μM). Most derivatives (**1**, **2**, **8**, **9**, **10**) demonstrated mild cytotoxic properties (CC_50_ = 28.6 ± 0.87 μM, 68 ± 0.59 μM, 85.9 ± 0.44 μM, 46.1 ± 0.76 μM, and 94 ± 0.79 μM, respectively). Camphecene exhibited low cytotoxicity (CC_50_ = 950.2 ± 0.25 μM). The reference drug umifenovir was shown to have mild cytotoxicity (CC_50_ = 27 ± 0.97 μM). For raw data see Supplementary Tables S5 and S6.

### 2.5. Evaluation of Activity Against SARS-CoV-2 Spike Protein 

To evaluate pseudovirus-inhibiting activity of the synthetic compounds, their ability to reduce the virus-mediated luciferase activity was measured at different concentrations of compounds. Based on the results obtained, values of IC_50_ were calculated for each compound, i.e., the concentration resulting in lowering the luciferase signal intensity by 50% compared to the control without additives, as well as luciferase signal reduction at the highest concentration (1/2 CC_50_) used. Pseudovirus activity was estimated as the difference in the percentage of luciferase activity in wells with zero compound content and in wells with the highest concentration of the compound. The antiviral activity data are presented in Table 1. Compounds **1**, **2**, **9**, **10**, and camphecene exhibited low activity against spike-pseudotyped lentiviruses, with IC_50_ values of 6.6 ± 0.05 μM, 30 ± 2.95 μM, 23.8 ± 0.08 μM, 25 ± 2.24 μM, and 549.3 ± 21.7 μM, respectively. As for VSV-G-pseudotyped lentiviruses, compounds **4**, **10**, and umifenovir showed low activity with IC_50_ of 59.3 ± 0.20 μM, 20.0 ± 0.89 μM and 8.8 ± 0.13 μM, respectively. At the highest tested concentrations, spike-pseudotyped lentivirus activity was reduced by 45–98% compared to control wells, while VSV-G-pseudotyped lentivirus activity decreased by 14–99%. All other examined derivatives demonstrated no antiviral properties at the tested concentrations.

Selectivity index (SI) values were calculated for the compounds. It is widely accepted that compounds with an SI of 10 or higher are considered active against virus in vitro in a sufficient range of non-toxic concentrations. No compound among those tested exceeded this selectivity threshold (Table 1). For raw data see Supplementary Tables S7.1–S7.24.

## 3. Discussion

In this study, we aimed to develop a pseudovirus-based test system for screening compounds that inhibit spike-mediated SARS-CoV-2 entry. To achieve this goal, we sought to combine and improve the most effective approaches previously published. The developed test system demonstrates high efficacy in screening potential inhibitors targeting the SARS-CoV-2 spike (S) protein. A key advantage of this system is the use of the human, non-small-cell lung carcinoma cell line H1299 engineered to overexpress *ACE2* (H1299-hACE2). In our work, we performed a comparative analysis of the sensitivity of two lines (H1299 and H1299-hACE2) to infection by spike-pseudotyped lentivirus or with G-pseudotyped lentivirus (Figure 2C). Consistent with reported results [41,42], we noted that G-pseudotyped lentivirus infects cell lines H1299 and H1299-hACE2 equally effectively, unlike spike-pseudotyped lentivirus, which infects only H1299-hACE2. However, unlike previous studies, we showed that infection of H1299-hACE2 with spike-pseudotyped lentivirus is followed by a stronger luciferase signal than infection of H1299-hACE2 with G-pseudotyped lentivirus. Thereby, unlike the widely used models that primarily utilize HEK293T cells with *ACE2* overexpression (HEK293T-ACE2) [41,42], the H1299-hACE2 cell line exhibits stronger susceptibility to SARS-CoV-2 spike-pseudotyped lentiviral particles compared to those pseudotyped with VSV-G.

Importantly, the use of H1299-hACE2 cells provides greater physiological relevance since lung epithelium is the primary entry site for SARS-CoV-2. Previous studies on SARS-CoV (the closest known relative of SARS-CoV-2) have shown that abundant *ACE2* expression in lung tissue facilitates viral entry [43]. In this context, H1299-hACE2 cells offer a valuable model, not only for screening antiviral agents, but also for isolating and propagating new strains of SARS-CoV-2.

Previously, Khan et al. [44] used H1299-ACE2 cells to study the SARS-CoV-2 BA.2.86 subvariant in their work, but the method for obtaining this cell line was not described in enough detail. Salgado-Benvindo et al. [45] generated H1299/ACE2 cells via transient transfection of H1299 cells and also utilized them to study different strains of SARS-CoV-2. For the first time within a single study, the susceptibility of the stable H1299-ACE2 cell line towards a broad panel of SARS-CoV-2 strains including Wuhan, Beta, Delta and Omicron was assessed.

In this study, we describe in detail the method of obtaining H1299 cells with *ACE2* overexpression by lentiviral transgenesis. The resulting H1299-hACE2 cell line was shown to be highly susceptible to infection by authentic SARS-CoV-2 (Wuhan strain) and spike-pseudotyped lentiviruses. We showed that the obtained stable H1299-hACE2 cells are effectively infected by SARS-CoV-2 (Wuhan strain 17612, Wuhan strain 18446, Beta, Delta and Omicron) and spike-pseudotyped lentiviruses. We subsequently employed this cell line in a pseudovirus-based assay to screen for inhibitors of the SARS-CoV-2 spike protein.

Notably, the H1299-hACE2 cell line we developed was generated through lentiviral transgenesis, ensuring stable and consistent *ACE2* expression. This contrasts with transient transfection, where the transfecting plasmid is gradually lost during cell division, leading to a progressive decline in *ACE2* expression levels over passages.

Upon infection with SARS-CoV-2, H1299-hACE2 cells form multinucleated syncytia, a cytopathic effect consistent with histopathological findings in lung tissues of COVID-19 patients [46]. This further supports the applicability of the model for studying virus-induced cytopathic effects. Overall, the H1299-hACE2 cell system closely replicates key aspects of viral entry and pathogenesis. This offers a robust platform for both basic research and drug development efforts targeting SARS-CoV-2.

In addition, our assay incorporates a reporter system featuring an enhanced *luc+* version of the firefly luciferase gene, resulting in greater sensitivity and a higher signal-to-noise ratio. Furthermore, with relatively simple manipulations, this cell line for the luciferase-based assay can be transformed EGFP- containing cells, the line that is more suitable for morphological and visual assays.

The discovery of low-molecular-weight entry and fusion inhibitors represents a promising approach for novel antivirals development. Indeed, various drugs effective against enveloped viruses have been created, such as the anti-influenza drug umifenovir (Arbidol) [47]; the anti-HIV drugs enfuvitide (T-20), temsavir, and its progenitor fostemsavir [48]; and the anti-RSV compounds sisunatovir and ziresovir [49]. Cell-based assays using fully infective viruses are often limited in drug development due to safety reasons. In this context, the use of non-dangerous viruses pseudotyped with surface proteins of highly pathogenic viruses is a good alternative approach for two reasons. First, it eliminates the risk of infecting personnel. Second, it simplifies questions related to the target and the inhibitor’s mechanism of action.

In our study, we employed a pseudovirus-based assay to evaluate the virus-suppressing properties of chemical compounds that are related to spike protein-mediated entry and fusion inhibition. The chemical library in the study was rather limited, and few compounds demonstrated a high level of antiviral activity. No morphological signs of cell toxicity or strong differences in infected cell morphology were observed at non-cytotoxic concentrations of any compound after visual evaluation. However, a positive correlation was observed between the values of SI and the extent of luciferase activity inhibition. Indeed, compounds leading to reduced pseudovirus activity by more than by 50% (e.g., **1**, **10**) possessed higher SI values.

Camphecene, a compound previously shown to inhibit influenza virus replication [50], inhibited spike protein mediated fusion by 74%. However, it demonstrated negligible activity against spike-pseudotyped lentiviruses (IC50 = 549.3 µM, SI = 2) and against VSV-G-pseudotyped lentiviruses (IC50 > 181.6 µM, SI < 5). This was likely due to its low bioavailability in the cell line we used. Umifenovir was previously shown to possess moderate activity against SARS-CoV-2 by CPE reduction assay (CC50 = 106 µM, IC50 23.6–29.0 µM, SI = 3–4) [51], yet was inactive by pseudovirus-based assay against spike-pseudotyped lentiviruses (CC50 = 14.8 µM, IC50 = 7.8 µM, SI = 2) [52]. The latter result matches our findings. Additionally, umifenovir showed moderate activity against VSV-G-pseudotyped lentiviruses (CC50 = 27 ± 0.97 µM, IC50 = 8.8 ± 0.13 µM, SI = 3), which may indicate anti-VSV-G activity.

In contrast, compounds **1** and **2**, which were reported as active in a previous study mentioned (SI = 17 and SI = 10, respectively), showed limited activity in our assay (SI = 4 and SI = 2, respectively), although both compounds resulted in about 50% inhibition of fusion activity. There are several explanations for this: the different cell lines used in the two studies; possible differences in drug activity by cell type; or potentially higher pseudovirus activity by cell type (requiring higher doses of inhibitor for suppression). With the exception of compound **10**, none of the sclareol derivatives exhibited significant activity. Compound **10** showed similar activities against spike-pseudotyped lentiviruses (IC50 = 25 ± 2.24 µM, SI = 4) and against VSV-G-pseudotyped lentiviruses (IC50 > 20 ± 0.89 µM, SI = 5), which could be a result of non-specific antiviral activity targeted on the cellular proteins, luciferase or on the proteins of the lentiviral backbone.

The coronavirus spike protein is essential for viral replication because it possesses two key functions. First, it mediates viral attachment to the host cell surface by binding to the ACE2 receptor. Second, following structural rearrangement, it drives fusion between the viral envelope and the host plasma membrane. Strictly speaking, definite conclusions cannot me made about whether a compound blocks the processes of viral attachment or membrane fusion based on our luciferase inhibition results. While compounds showing inhibitory activity in our pseudovirus-based assay are likely entry inhibitors, but their precise molecular target and mechanism of action would require further investigation. The observed reduction in luciferase signal could also result from non-specific effects, such as overall transcription and translation inhibition, as well as the lentiviral backbone or the reporter enzyme inhibition. The use of a VSV-G-pseudotyped control helps eliminate some false positives arising from such non-specific mechanisms. Consequently, a more detailed study of the active compounds identified here is necessary to elucidate their exact mechanism of action.

The developed pseudoviral platform has certain limitations stemming from the replicative inactivity of the lentiviral backbone. Consequently, pseudoviruses cannot model the full viral replication cycle or support many classical virological techniques, including virus yield reduction assays, plaque reduction assays, and methods for quantifying viral genomes. Despite this, the replicative inactivity of the lentiviral backbone brings an essential advantage: this characteristic makes pseudoviral platforms safe for use in standard BSL-2 biosafety laboratories.

## 4. Materials and Methods

### 4.1. Cell Lines and Viruses

Human embryonic kidney cells (HEK293T) and human lung carcinoma cells (H1299) were obtained from the Saint Petersburg Pasteur Institute cell line collection. Cells were cultivated (37 °C, 5% CO_2_) in Dulbecco’s modified Eagle’s medium F12 (DMEM/F12 (1:1)) with L-glutamine (BioloT, Moscow, Russia), 10% fetal bovine serum (Gibco, London, UK), 100 units/mL penicillin, 0.1 mg/mL streptomycin sulfate, and 25 μg/mL amphotericin B (Himedia, Maharashtra, India).

To obtain target cell line H1299-hACE2, which is highly permissive to SARS-CoV-2 virus, we performed the following. H1299 cells (70% confluence) were transduced with dHIV-VSV-G-hACE2 lentivirus containing human *ACE2* cDNA under control of the EF-1α promoter. Cells were incubated for 48 h, after which the selective antibiotic puromycin (2 μg/mL) was added. Selection was performed for one week.

The relative expression of *ACE2* was analyzed using qRT-PCR and the 2^−ΔΔC^_T_ method [53]. Total RNA was extracted from H1299 and H1299-hACE2 cell lines using ExtractRNA reagent (Evrogen, Moscow, Russia) according to the manufacturer’s protocol. cDNA synthesis was performed with random hexamer primers using the RevertAid RT Reverse Transcription Kit (Thermo Fisher Scientific, Waltham, MA, USA) according to the manufacturer’s protocol. Reactions (qRT-PCR) were performed on the CFX96 Touch Real-Time PCR Detection System (Bio-Rad, USA). All reactions were run in triplicate using primers for *ACE2* (forward CCCTGCTCATTTGCTTGCTTGGTGTG, reverse ACATTTCCTGTCCGTCCGTTAGC) and the reference gene (GAPDH).

SARS-CoV-2 virus strains Wuhan 17612, Wuhan 18446, Beta, Delta and Omicron were obtained from the viral collection of the Pasteur Institute (St. Petersburg, Russia). A functional test of the obtained H1299-hACE2 cell line was also performed. H1299 and H1299-hACE2 cell lines were infected with different strains of SARS-CoV-2 virus and cell morphology was visually analyzed 24 h post-infection using the Cytation 5 device (Biotek, Miami, FL, USA).

### 4.2. Plasmids

To obtain lentiviral transfer plasmid pCDH-EF1-FHC-hACE2 encoding the human ACE2 gene, total RNA was extracted from cell line H1299 using ExtarctRNA reagent (Evrogen, Russia). cDNA synthesis was performed with Oligo(dT)18 primer using the RevertAid RT Reverse Transcription Kit (Thermo Fisher Scientific, Waltham, MA, USA) according to the manufacturer’s protocol. The *ACE2* gene (NM_001371415.1) was amplified with forward (AATCTAGACGATGTCAAGCTCT) and reverse (AAGCTAGCAATCACCTCAAGAGAGGAA) primers and cloned into pCDH-EF1-FHC plasmid (Addgene plasmid #64874) by XbaI and NheI restriction sites.

To obtain the lentiviral transfer plasmid pCDH-EF1-FHC-Luc-ΔIRES-Puro, the enhanced *luc+* version of the firefly luciferase gene from plasmid pGL3-Basic (Promega, Madison, WI, USA) was amplified with forward (AAAAAGCTAGCGGTAAAGCCACCATGGAAGAC) and reverse (TGACAGTCGACAGAATTACACGGCGATCTTTCC) primers and recloned into pCDH-EF1-FHC plasmid by NheI and SalI restriction sites.

Plasmid pMD2-MCS was derived from plasmid pMD2.G (Addgene plasmid # 12259) by replacing the *VSV-G* gene with multiple cloning sites. A codon-optimized SARS-CoV-2 *spike* gene (Wuhan strain) was obtained from plasmid pCG1-SARS-2-S-Sirius [54]. It was then amplified using forward (ATAAAGCTAGCCACCACCATGTTCGTTCGTGTTTCTGGTG) and reverse (ATAATCTCGAGTCGCGCGACTTAAGTTAGTTAGGTGTGTAGTAGTG) primers, and re-cloned (NheI and XhoI restriction sites) into the plasmid pMD2-MCS. The obtained envelope plasmid was named pMD2-optSpike.

**Figure 3:**
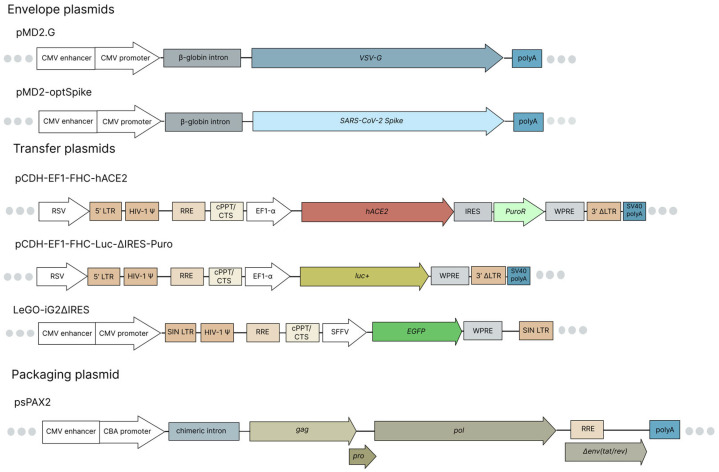
Maps of plasmids used. The envelope plasmids pMD2.G and pMD2-optSpike encode the *VSV-G* and the codon-optimized *spike* genes, respectively, under the control of the CMV enhancer/promoter with the β-globin intron. The lentiviral transfer plasmid pCDH-EF1-FHC-hACE2 encodes the human *ACE2* gene under control of the EF1-alpha promoter. The lentiviral transfer plasmid pCDH-EF1-FHC-Luc-ΔIRES-Puro carries an enhanced *luc+* gene under the EF1-alpha promoter. To enhance lentivirus assembly efficiency, the IRES and PuroR elements were removed. The lentiviral transfer plasmid LeGO-iG2ΔIRES encodes *EGFP* under control of the SFFV promoter. The packaging plasmid psPAX2 encodes the *gag*, *pol*, and *rev* genes essential for lentivirus packaging.

The pCG1-SARS-2-S-Sirius plasmid was kindly by provided Prof. Dr. Sergey Nedospasov and Dr. Irina Astrakhantseva (Sirius University of Science and Technology, Sochi, Russia). The LeGO-iG2ΔIRES plasmid, encoding *EGFP* under the control of the SFFV promoter, was kindly provided by Alexey Petukhov (Nazarbayev University School of Medicine, Astana, Kazakhstan). Maps of the plasmids used in this study are shown in Figure 3.

### 4.3. Pseudotyped Virus Production

HEK293T cells were seeded onto a 6-well plate (8 × 10^5^ cells/well) using DMEM/F12 culture medium (BioloT, Moscow, Russia). Cells reached 70% confluence in 24 h of incubation (37 °C, 5% CO_2_). Culture media were then replaced with transfection mixture: 0.1 mg/mL PEI (Polysciences, Warrington, PA, USA) in optiMEM (Gibco, Norristown, PA, USA). The following DNA amounts were preliminary added to the 100 μg transfection mixture to assemble each recombinant lentivirus (LV) shown below (Table 2): 500 ng envelope plasmid; 1000 ng packaging plasmid psPAX2 (Addgene plasmid #12260); and 1250 ng transfer plasmid. Growth medium was changed 24 h after transfection, and cells were incubated for 48 h (37 °C, 5% CO_2_). Medium with recombinant LV was collected, purified by centrifugation (5 min, 1000 rpm), and filtered through syringe filters (0.45 μm PES). Lentivirus concentrates were aliquoted and frozen at −80 °C.

### 4.4. Transduction

For the fluorescence-based assay, a 70% confluent monolayer of H1299 or H1299-hACE2 cells in black-well plates was transduced with either dHIV-VSV-G-EGFP or dHIV-SARS-2-S-EGFP recombinant lentivirus. Cells were then incubated for 48 h (37 °C, 5% CO_2_). After transduction, cell morphology was analyzed visually, and level of fluorescence was measured using Cytation 5 cell imaging multimode reader (Biotek, Miami, FL, USA).

For the luciferase-based assay, a 70% confluent monolayer of H1299 or H1299-hACE2 cells in white-well plates was transduced with either dHIV-VSV-G-Luc or dHIV-SARS-2-S-Luc LVs recombinant lentivirus. Cells were incubated for 48 h (37 °C, 5% CO_2_). Luciferase activity was analyzed 48 h after transduction using the AbiLux Firefly Luciferase Assay Kit (Abisense, Moscow, Russia) according to the manufacturer’s protocol and the Varioskan LUX multimode microplate reader (Thermo Fisher Scientific, Waltham, MA, USA).

### 4.5. Electron Microscopy

Before freezing, a drop of suspension (see section “Pseudotyped virus production”) was placed on a Petri dish. A carbon-coated EM grid was placed on top of the drop for 2 min. After removing the virus-containing drop, the grid was washed with distilled water and placed onto a drop of 2.0% phosphotungstic acid (pH 7.0) for one minute. Excess phosphotungstic acid was carefully removed with filter paper, and the EM grid was examined in a JEM-100S electron microscope (JEOL, Tokyo, Japan) at 30,000–150,000 magnification.

### 4.6. Compounds

Compounds for the study were synthetized by the Vorozhtsov Novosibirsk Institute of Organic Chemistry. Synthesis of compounds **1** and **2** is described by Yarovaya et al. (2022) [52]. Ester derivatives of (−)-borneol **1** and **2** are active as entry inhibitors of respiratory syncytial virus [55], filoviruses [56] and influenza viruses [57]. It has been shown that (−)-Sclareol (**10**) and Sclareolide (**3**) are active as effective inhibitors of filovirus entry [58]. 8-Epi-sclareolide (**4**) was obtained by the previously described procedure [59]. (+)-Amberketal (**5**) was obtained by the method described in the work [60]. The synthesis of labdan-type olefin **6**, labdanediol (**7**), 13-epi-sclareol (**8**) and diol **9** is described in the work [61]. Sclareol and its derivatives are widely used in the synthesis of natural compounds [62] and have a wide range of biological activity [63]. Camphecene is active against a wide range of influenza viruses as entry inhibitor [50]. Umifenovir was used as a comparison drug [64].

### 4.7. Cytotoxicity Evaluation Method

To evaluate any toxic effects of compounds on cells, 50% cytotoxic concentration (CC50) values were determined. To define CC50, H1299-hACE2 cells were seeded onto 96-well plates (10^4^ cells per well) and incubated for 24 h (37 °C, 5% CO_2_). Next, compounds were added to wells in a series of three-fold dilutions (60 μg/mL, 20 μg/mL, 6.6 μg/mL, 2.2 μg/mL, 0.74 μg/mL, 0.25 μg/mL) in duplicate. Cells were incubated with compounds for 1 h (37 °C, 5% CO_2_). Afterwards, an equal volume of media was added to each well (further diluting initial concentrations two-fold). Plates were then incubated for 48 h (37 °C, 5% CO_2_). MTT assays were performed using tetrazolium bromide. For this purpose, the cells were washed 2 times with saline (0.9% NaCl), and 100 μL/well of MTT solution [3-(4,5-dimethylthiazol-2-yl)-2,5-diphenyltetrazolium bromide, BioFroxx, Germany] at a concentration of 0.5 mg/mL in MEM was added. The plates were incubated for 1 h at 36 °C, the liquid was removed, and dimethylsulfoxide (DMSO) (0.1 mL per well) was added. Further, optical density values were measured using the Multiscan FC instrument (Thermo Fisher Scientific, Waltham, MA, USA), and CC50 values were calculated via GraphPad Prism 8 (San Diego, CA, USA). For final presentation, CC_50_ values in μg/mL were calculated into micromoles.

### 4.8. Evaluation of Activity Against SARS-CoV-2 Spike Protein Method

To define the effects of compounds on SARS-CoV-2 spike protein, the 50% inhibitory concentration (IC_50_) and percent pseudovirus activity values were determined. To achieve this, H1299-hACE2 cells were seeded onto 96-well plates (10^4^ cells/well) and incubated for 24 h (37 °C, 5% CO_2_). A series of three-fold compound dilutions were then prepared (CC_50_/2, CC_50_/6, CC_50_/18, CC_50_/54) and added to cells in six replicates, followed by incubation for 1 h (37 °C, 5% CO_2_). Next, cells were infected with SARS-CoV-2 spike or VSV-G-pseudotyped lentiviral particles with *luc+* reporter gene (dHIV-SARS-2-S-Luc and HIV-SARS-2-S-Luc) and incubated for 48 h (37 °C, 5% CO_2_). Finally, luciferase activity was determined using the AbiLux Firefly Luciferase Assay Kit (Abisense, Moscow, Russia) and the Varioskan LUX Multimode Microplate Reader (Thermo Fisher Scientific, Waltham, MA, USA). From the obtained data, IC50 and percentage of pseudovirus activity were calculated. For final presentation, IC_50_ values in μg/mL were calculated into micromoles.

### 4.9. Data Analysis

Data were analyzed via GraphPad Prism 8 Software (San Diego, CA, USA). Two-tailed Student’s *t*-test was used to compare the ACE2 expression (ΔCt) between H1299 and H1299-hACE2 cell lines. The luciferase activity and fluorescence intensity of the same cell lines were analyzed with the Mann–Whitney U test. CC_50_ and IC_50_ values were calculated using a nonlinear regression curve fit to data expressed as % inhibition of infection.

## 5. Conclusions

In the present study, we have developed and characterized a luciferase-based assay using SARS-CoV-2 spike-pseudotyped lentivirus and the H1299-hACE2 cell line. The stable and consistent *ACE2* expression in this engineered cell line eliminates the risk of potential loss of transfecting plasmid(s) inherent to transient transfected cell lines. This property also makes H1299-hACE2 cells highly permissive to SARS-CoV-2 infection, a feature applicable to diverse uses ranging from primary virus isolation to potential antiviral screening and antibody testing. Furthermore, because the parental H1299 cells are derived from lung epithelium—the primary site of SARS-CoV-2 entry—the H1299-hACE2 model offers greater physiological relevance than systems based on non-lung cell lines.

Using this assay, we assessed the virus-inhibiting activity of potential S protein blockers. We showed that derivatives of 1,7,7-trimethyl-[2.2.1]-bicycloheptane possess moderate activity against lentiviral particles. As such, these may represent promising precursors for potential antivirals. Moreover, the described lentiviral test system allows one to distinguish false-positive activity of compounds against a spike protein.

## Figures and Tables

**Table 1:** Cytotoxicity, antiviral activity, selectivity of compounds against SARS-CoV-2 S protein and VSV-G by pseudovirus-based assay. Presented are values for CC50 (μM, mean ± SD), IC50 (μM, mean ± SD), pseudovirus activity (%, mean ± SD), SI (CC50: IC50 ratio).

Compound No	Structure	Cytotoxicity	Antiviral Activity LV-Spike		Selectivity LV-Spike	Antiviral Activity LV-G		Selectivity LV-G
		CC_50_ (μM) *^a^*	IC_50_ (μM) *^b^*	Pseudovirus Activity (%) *^c^*	SI *^d^*	IC_50_ (μM) *^b^*	Pseudovirus Activity (%) *^c^*	SI *^d^*
**1**	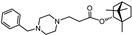	28.6 ± 0.87	6.6 ± 0.05	41.9 ± 4.12	4	>14.3	130.6 ± 0.43	<2
**2**	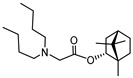	68 ± 0.59	30 ± 2.95	50 ± 3.77	2	>34.0	85.8 ± 1.79	<2
**3** (+)-Sclareolide	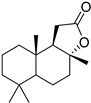	>119.8	>59.9	92.9 ± 0.12	<2	>59.9	119.9 ± 3.93	<2
**4** 8-epi-sclareolide	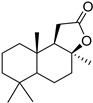	>119.8	>59.9	76.2 ± 1.77	<2	59.3 ± 0.20	46.9 ± 12.38	2
**5** Amberketal	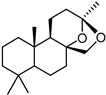	>107.7	>53.9	67.9 ± 6.07	<2	>53.9	111.4 ± 11.14	<2
**6**	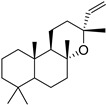	>103.3	>51.6	82.2 ± 4.26	<2	>51.6	101.4 ± 5.00	<2
**7** Labdanediol	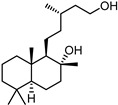	12.9 ± 1.03	>6.4	102.6 ± 2.29	<2	>6.4	109.0 ± 3.63	<2
**8** 13-*epi*-sclareol	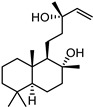	85.9 ± 0.44	>42.1	82.4 ± 4.21	<2	>42.1	111.7 ± 11.29	<2
**9**	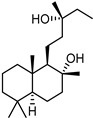	46.1 ± 0.76	23.8 ± 0.08	58 ± 4.74	2	>22.5	84.8 ± 0.22	<2
**10** (−)-Sclareol	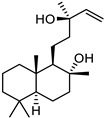	94 ± 0.79	25 ± 2.24	2.1 ± 0.15	4	20.0 ± 0.89	0.2 ± 0.09	5
camphecene	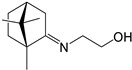	950.2 ± 0.25	549.3 ± 21.7	26 ± 0.15	2	>181.6	67.0 ± 2.28	<5
Umifenovir	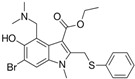	27 ± 0.97	>27	93.6 ± 3.66	1	8.8 ± 0.13	42.9 ± 0.04	3

Note: *^a^* CC_50_—50% cytotoxic concentration (resulting in death of 50% of cells in uninfected monolayers); *^b^* IC_50_—50% virus inhibitory concentration (resulting in protection of 50% of cells in infected monolayers); *^c^* level of luciferase signal (as percent of DMSO control); *^d^* SI—selectivity index (CC_50_/IC_50_ ratio).

**Table 2:** Plasmids used for assembly of recombinant lentiviruses.

Recombinant LV	Envelope Plasmid	Transfer Plasmid
dHIV-VSV-G-hACE2	pMD2.G (Addgene plasmid # 12259)	pCDH-EF1-FHC-hACE2
dHIV-VSV-G-EGFP	pMD2.G	LeGO-iG2ΔIRES
dHIV-VSV-G-Luc	pMD2.G	pCDH-EF1-FHC-Luc-ΔIRES-Puro
dHIV-SARS-2-S-EGFP	pMD2-optSpike	LeGO-iG2ΔIRES
dHIV-SARS-2-S-Luc	pMD2-optSpike	pCDH-EF1-FHC-Luc-ΔIRES-Puro

## Data Availability

Data can be obtained from the corresponding author by request.

## Supplementary Materials

The following are available online at https://www.mdpi.com/article/10.3390/ijms27020791/s1, Tables S1 and S2: raw data for gene expression analysis for ACE2 and GAPDH; Table S3: raw da-ta for fluorescence activity measurement for pseudoviruses dHIV-VSV-G, dHIV-SARS-2-S; Table S4: raw data for luciferase activity measurement for pseudoviruses dHIV-VSV-G, dHIV-SARS-2-S; Tables S5 and S6: raw data for compounds’ cytotoxicity assessment; Tables S7.1–S7.24: raw data for compounds’ anti-pseudovirus activity assessment; Figure S1: evaluation of CPE in cells infected with VSV-G and spike-pseudotyped lentiviruses.

## References

[1] Yang Y., Peng F., Wang R., Guan K., Jiang T., Xu G., Sun J., Chang C. (2020). The Deadly Coronaviruses: The 2003 SARS Pandemic and the 2020 Novel Coronavirus Epidemic in China. J. Autoimmun..

[2] Middle East Respiratory Syndrome Coronavirus (MERS-CoV). https://www.who.int/health-topics/middle-east-respiratory-syndrome-coronavirus-mers.

[3] COVID-19 Cases. WHO COVID-19 Dashboard. https://data.who.int/dashboards/covid19/cases.

[4] Klein S., Cortese M., Winter S.L., Wachsmuth-Melm M., Neufeldt C.J., Cerikan B., Stanifer M.L., Boulant S., Bartenschlager R., Chlanda P. (2020). SARS-CoV-2 Structure and Replication Characterized by in Situ Cryo-Electron Tomography. Nat. Commun..

[5] Fertig T.E., Chitoiu L., Terinte-Balcan G., Peteu V., Marta D., Gherghiceanu M. (2022). The Atomic Portrait of SARS-CoV-2 as Captured by Cryo-electron Microscopy. J. Cell. Mol. Med..

[6] Jackson C.B., Farzan M., Chen B., Choe H. (2022). Mechanisms of SARS-CoV-2 Entry into Cells. Nat. Rev. Mol. Cell Biol..

[7] Williams R., Hales J., Collier W., Gould P. (2025). Coronavirus Replication: Genomes, Subgenomic RNAs, and Defective Viral Genomes. Viruses.

[8] V’kovski P., Kratzel A., Steiner S., Stalder H., Thiel V. (2021). Coronavirus Biology and Replication: Implications for SARS-CoV-2. Nat. Rev. Microbiol..

[9] Shum M.H.-H., Lee Y., Tam L., Xia H., Chung O.L.-W., Guo Z., Lam T.T.-Y. (2024). Binding Affinity between Coronavirus Spike Protein and Human ACE2 Receptor. Comput. Struct. Biotechnol. J..

[10] Everest H., Stevenson-Leggett P., Bailey D., Bickerton E., Keep S. (2022). Known Cellular and Receptor Interactions of Animal and Human Coronaviruses: A Review. Viruses.

[11] Raj V.S., Mou H., Smits S.L., Dekkers D.H.W., Müller M.A., Dijkman R., Muth D., Demmers J.A.A., Zaki A., Fouchier R.A.M. (2013). Dipeptidyl Peptidase 4 Is a Functional Receptor for the Emerging Human Coronavirus-EMC. Nature.

[12] Yang Y., Du L., Liu C., Wang L., Ma C., Tang J., Baric R.S., Jiang S., Li F. (2014). Receptor Usage and Cell Entry of Bat Coronavirus HKU4 Provide Insight into Bat-to-Human Transmission of MERS Coronavirus. Proc. Natl. Acad. Sci. USA.

[13] Song W., Gui M., Wang X., Xiang Y. (2018). Cryo-EM Structure of the SARS Coronavirus Spike Glycoprotein in Complex with Its Host Cell Receptor ACE2. PLoS Pathog..

[14] Wang Q., Zhang Y., Wu L., Niu S., Song C., Zhang Z., Lu G., Qiao C., Hu Y., Yuen K.-Y. (2020). Structural and Functional Basis of SARS-CoV-2 Entry by Using Human ACE2. Cell.

[15] Cui J., Li F., Shi Z.-L. (2019). Origin and Evolution of Pathogenic Coronaviruses. Nat. Rev. Microbiol..

[16] Pekar J.E., Lytras S., Ghafari M., Magee A.F., Parker E., Wang Y., Ji X., Havens J.L., Katzourakis A., Vasylyeva T.I. (2025). The Recency and Geographical Origins of the Bat Viruses Ancestral to SARS-CoV and SARS-CoV-2. Cell.

[17] Holmes E.C., Goldstein S.A., Rasmussen A.L., Robertson D.L., Crits-Christoph A., Wertheim J.O., Anthony S.J., Barclay W.S., Boni M.F., Doherty P.C. (2021). The Origins of SARS-CoV-2: A Critical Review. Cell.

[18] Li Z., Jiang J., Ruan X., Tong Y., Xu S., Han L., Xu J. (2021). The Zoonotic and Natural Foci Characteristics of SARS-CoV-2. J. Biosaf. Biosecurity.

[19] Nie J., Li Q., Zhang L., Cao Y., Zhang Y., Li T., Wu J., Liu S., Zhang M., Zhao C. (2021). Functional Comparison of SARS-CoV-2 with Closely Related Pangolin and Bat Coronaviruses. Cell Discov..

[20] Wrobel A.G., Benton D.J., Xu P., Roustan C., Martin S.R., Rosenthal P.B., Skehel J.J., Gamblin S.J. (2020). SARS-CoV-2 and Bat RaTG13 Spike Glycoprotein Structures Inform on Virus Evolution and Furin-Cleavage Effects. Nat. Struct. Mol. Biol..

[21] Delgado J.M., Duro N., Rogers D.M., Tkatchenko A., Pandit S.A., Varma S. (2021). Molecular Basis for Higher Affinity of SARS-CoV-2 Spike RBD for Human ACE2 Receptor. Proteins.

[22] Piplani S., Singh P.K., Winkler D.A., Petrovsky N. (2021). In Silico Comparison of SARS-CoV-2 Spike Protein-ACE2 Binding Affinities across Species and Implications for Virus Origin. Sci. Rep..

[23] Mohamed K., Rzymski P., Islam M.S., Makuku R., Mushtaq A., Khan A., Ivanovska M., Makka S.A., Hashem F., Marquez L. (2022). COVID-19 Vaccinations: The Unknowns, Challenges, and Hopes. J. Med. Virol..

[24] Shah S.K., Bhandari K., Shah A., Chaurasiya G. (2024). COVID-19: Vaccination, Therapeutics and a Review of the Science and Public Health. Ann. Med. Surg..

[25] Rajanala K., Upadhyay A.K. (2024). Vaccines for Respiratory Viruses—COVID and Beyond. Vaccines.

[26] Cui W., Duan Y., Gao Y., Wang W., Yang H. (2024). Structural Review of SARS-CoV-2 Antiviral Targets. Structure.

[27] Mawazi S.M., Fathima N., Mahmood S., Al-Mahmood S.M.A. (2024). Antiviral Therapy for COVID-19 Virus: A Narrative Review and Bibliometric Analysis. Am. J. Emerg. Med..

[28] Terwilliger E.F., Godin B., Sodroski J.G., Haseltine W.A. (1989). Construction and Use of a Replication-Competent Human Immunodeficiency Virus (HIV-1) That Expresses the Chloramphenicol Acetyltransferase Enzyme. Proc. Natl. Acad. Sci. USA.

[29] Page K.A., Landau N.R., Littman D.R. (1990). Construction and Use of a Human Immunodeficiency Virus Vector for Analysis of Virus Infectivity. J. Virol..

[30] Landau N.R., Page K.A., Littman D.R. (1991). Pseudotyping with Human T-Cell Leukemia Virus Type I Broadens the Human Immunodeficiency Virus Host Range. J. Virol..

[31] Akkina R.K., Walton R.M., Chen M.L., Li Q.X., Planelles V., Chen I.S. (1996). High-Efficiency Gene Transfer into CD34+ Cells with a Human Immunodeficiency Virus Type 1-Based Retroviral Vector Pseudotyped with Vesicular Stomatitis Virus Envelope Glycoprotein G. J. Virol..

[32] Reiser J., Harmison G., Kluepfel-Stahl S., Brady R.O., Karlsson S., Schubert M. (1996). Transduction of Nondividing Cells Using Pseudotyped Defective High-Titer HIV Type 1 Particles. Proc. Natl. Acad. Sci. USA.

[33] Zhang L., Liu S., Wang Y., Wang Y. (2023). Pseudotyped Viruses for Marburgvirus and Ebolavirus. Pseudotyped Viruses.

[34] Yang R., Huang B., A. R., Li W., Wang W., Deng Y., Tan W. (2020). Development and Effectiveness of Pseudotyped SARS-CoV-2 System as Determined by Neutralizing Efficiency and Entry Inhibition Test in Vitro. Biosaf. Health.

[35] Schmidt F., Weisblum Y., Muecksch F., Hoffmann H.-H., Michailidis E., Lorenzi J.C.C., Mendoza P., Rutkowska M., Bednarski E., Gaebler C. (2020). Measuring SARS-CoV-2 Neutralizing Antibody Activity Using Pseudotyped and Chimeric Viruses. J. Exp. Med..

[36] Johnson M.C., Lyddon T.D., Suarez R., Salcedo B., LePique M., Graham M., Ricana C., Robinson C., Ritter D.G. (2020). Optimized Pseudotyping Conditions for the SARS-COV-2 Spike Glycoprotein. J. Virol..

[37] Fu X., Tao L., Zhang X. (2021). Comprehensive and Systemic Optimization for Improving the Yield of SARS-CoV-2 Spike Pseudotyped Virus. Mol. Ther. Methods Clin. Dev..

[38] Calder L.J., Calcraft T., Hussain S., Harvey R., Rosenthal P.B. (2022). Electron Cryotomography of SARS-CoV-2 Virions Reveals Cylinder-Shaped Particles with a Double Layer RNP Assembly. Commun. Biol..

[39] Kordyukova L.V., Moiseenko A.V., Serebryakova M.V., Shuklina M.A., Sergeeva M.V., Lioznov D.A., Shanko A.V. (2023). Structural and Immunoreactivity Properties of the SARS-CoV-2 Spike Protein upon the Development of an Inactivated Vaccine. Viruses.

[40] Schoderboeck L., Riad S., Bokor A.M., Wicky H.E., Strauss M., Bostina M., Oswald M.J., Empson R.M., Hughes S.M. (2015). Chimeric Rabies SADB19-VSVg-Pseudotyped Lentiviral Vectors Mediate Long-Range Retrograde Transduction from the Mouse Spinal Cord. Gene Ther..

[41] Crawford K.H.D., Eguia R., Dingens A.S., Loes A.N., Malone K.D., Wolf C.R., Chu H.Y., Tortorici M.A., Veesler D., Murphy M. (2020). Protocol and Reagents for Pseudotyping Lentiviral Particles with SARS-CoV-2 Spike Protein for Neutralization Assays. Viruses.

[42] Hu J., Gao Q., He C., Huang A., Tang N., Wang K. (2020). Development of Cell-Based Pseudovirus Entry Assay to Identify Potential Viral Entry Inhibitors and Neutralizing Antibodies against SARS-CoV-2. Genes Dis..

[43] Hamming I., Timens W., Bulthuis M.L.C., Lely A.T., Navis G.J., van Goor H. (2004). Tissue Distribution of ACE2 Protein, the Functional Receptor for SARS Coronavirus. A First Step in Understanding SARS Pathogenesis. J. Pathol..

[44] Khan K., Lustig G., Römer C., Reedoy K., Jule Z., Karim F., Ganga Y., Bernstein M., Baig Z., Jackson L. (2023). Evolution and Neutralization Escape of the SARS-CoV-2 BA.2.86 Subvariant. Nat. Commun..

[45] Salgado-Benvindo C., Tas A., Zevenhoven-Dobbe J.C., van der Meer Y., Sidorov I.A., Leijs A.A., Wanningen P., Gelderloos A.T., van Kasteren P.B., Snijder E.J. (2024). Characterization of SARS-CoV-2 Replication in Human H1299/ACE2 Cells: A Versatile and Practical Infection Model for Antiviral Research and Beyond. Antiviral Res..

[46] Bussani R., Schneider E., Zentilin L., Collesi C., Ali H., Braga L., Volpe M.C., Colliva A., Zanconati F., Berlot G. (2020). Persistence of Viral RNA, Pneumocyte Syncytia and Thrombosis Are Hallmarks of Advanced COVID-19 Pathology. EBioMedicine.

[47] Kang Y., Shi Y., Xu S. (2023). Arbidol: The Current Demand, Strategies, and Antiviral Mechanisms. Immun. Inflamm. Dis..

[48] Huang Y., Liang G., Wang T., Ma Y., Ga L., Sun L., Qi X., Zhang W., Li R., Zhao Y. (2025). Research Strategies of the N-Peptide Fusion Inhibitor: A Promising Direction for Discovering Novel Antivirals. J. Virol..

[49] Bonneux B., Jacoby E., Ceconi M., Stobbelaar K., Delputte P., Herschke F. (2024). Direct-Acting Antivirals for RSV Treatment, a Review. Antiviral Res..

[50] Zarubaev V.V., Garshinina A.V., Tretiak T.S., Fedorova V.A., Shtro A.A., Sokolova A.S., Yarovaya O.I., Salakhutdinov N.F. (2015). Broad Range of Inhibiting Action of Novel Camphor-Based Compound with Anti-Hemagglutinin Activity against Influenza Viruses in Vitro and in Vivo. Antiviral Res..

[51] Leneva I., Kartashova N., Poromov A., Gracheva A., Korchevaya E., Glubokova E., Borisova O., Shtro A., Loginova S., Shchukina V. (2021). Antiviral Activity of Umifenovir In Vitro against a Broad Spectrum of Coronaviruses, Including the Novel SARS-CoV-2 Virus. Viruses.

[52] Yarovaya O.I., Shcherbakov D.N., Borisevich S.S., Sokolova A.S., Gureev M.A., Khamitov E.M., Rudometova N.B., Zybkina A.V., Mordvinova E.D., Zaykovskaya A.V. (2022). Borneol Ester Derivatives as Entry Inhibitors of a Wide Spectrum of SARS-CoV-2 Viruses. Viruses.

[53] Livak K.J., Schmittgen T.D. (2001). Analysis of Relative Gene Expression Data Using Real-Time Quantitative PCR and the 2^−ΔΔCT^ Method. Methods.

[54] Kruglova N., Siniavin A., Gushchin V., Mazurov D. (2021). Different Neutralization Sensitivity of SARS-CoV-2 Cell-to-Cell and Cell-Free Modes of Infection to Convalescent Sera. Viruses.

[55] Sokolova A.S., Yarovaya O.I., Kuzminykh L.V., Shtro A.A., Klabukov A.M., Galochkina A.V., Nikolaeva Y.V., Petukhova G.D., Borisevich S.S., Khamitov E.M. (2022). Discovery of N-Containing (-)-Borneol Esters as Respiratory Syncytial Virus Fusion Inhibitors. Pharmaceuticals.

[56] Sokolova A.S., Yarovaya O.I., Zybkina A.V., Mordvinova E.D., Shcherbakova N.S., Zaykovskaya A.V., Baev D.S., Tolstikova T.G., Shcherbakov D.N., Pyankov O.V. (2020). Monoterpenoid-Based Inhibitors of Filoviruses Targeting the Glycoprotein-Mediated Entry Process. Eur. J. Med. Chem..

[57] Sokolova A.S., Yarovaya O.I., Semenova M.D., Shtro A.A., Orshanskaya I.R., Zarubaev V.V., Salakhutdinov N.F. (2017). Synthesis and in Vitro Study of Novel Borneol Derivatives as Potent Inhibitors of the Influenza A Virus. MedChemComm.

[58] Chen Q., Tang K., Guo Y. (2020). Discovery of Sclareol and Sclareolide as Filovirus Entry Inhibitors. J. Asian Nat. Prod. Res..

[59] McCarthy F.O., Saarinen J., Upadhyay A., Zanetti C., Collins E.A., O’Driscoll M., Lynch O.M., Cronin M.F., Rüffer T., Antoniuk O. (2025). Facile Epimerization of (+)-Sclareolide: A Versatile Experiment for Laboratory Education. J. Chem. Educ..

[60] Martres P., Perfetti P., Zahra J.-P., Waegell B., Giraudi E., Petrzilka M. (1993). A Short and Practical Synthesis of (+)-Amberketal and (-)-Epi-8-Amberketal from Natural (-)-Sclareol. Tetrahedron Lett..

[61] Vlad P.F., Ungur N.D. (1983). New Convenient Methods for the Preparation of Tetrahydrofurans from 1,4-Diols. Synthesis.

[62] Selka A., Abidli A., Schiavo L., Jeanmart L., Hanquet G., Lubell W.D. (2025). Recent Advances in Sustainable Total Synthesis and Chiral Pool Strategies with Emphasis on (−)-Sclareol in Natural Products Synthesis. Eur. J. Org. Chem..

[63] Tang H., Li L., Yu Q., Chen L., Xu X., Meng Z., Zeng Y., Chen F., Muzaffar H., Wang W. (2025). Sclareol Improves the Pathology of Alzheimer’s Disease by Inhibiting Microglial Inflammation via Interacting with CDK9. Phytomedicine Int. J. Phytother. Phytopharm..

[64] Borisevich S.S., Khamitov E.M., Gureev M.A., Yarovaya O.I., Rudometova N.B., Zybkina A.V., Mordvinova E.D., Shcherbakov D.N., Maksyutov R.A., Salakhutdinov N.F. (2022). Simulation of Molecular Dynamics of SARS-CoV-2 S-Protein in the Presence of Multiple Arbidol Molecules: Interactions and Binding Mode Insights. Viruses.

