# Supplementary material for: Development and Application of a Pseudovirus-Based Assay for Modelling SARS-CoV-2 Spike Protein Mediated Drug Screening
Source: Int J Mol Sci. 2026 Jan 13;27(2):791. doi: 10.3390/ijms27020791 (PMC12841467; doi:10.3390/ijms27020791)
Supplement: Supplementary file 1 [file ijms-27-00791-s001.zip › ijms-4037006-supplementary.pdf]

Development and Application of a Pseudovirus-Based Assay for Modelling SARS-CoV-2 Spike Protein Mediated Drug Screening

Shokhrukh A. Khasanov [1], Iana L. Esaulkova [1,*], Alexandrina S. Volobueva [1], Alexander V. Slita [2], Daria V. Kriger [3], Dmitri Tentler [4], Olga I. Yarovaya [5], Anastasia S. Sokolova [5], Andrey N. Gorshkov [6], Anna S. Dolgova [1], Irina N. Lavrentieva [1], Vladimir G. Dedkov [1,7], Nariman F. Salakhutdinov [5] and Vladimir V. Zarubaev [1]

[1] Saint Petersburg Pasteur Institute, Federal Service for the Oversight of Consumer Protection and Welfare, 197101 Saint Petersburg, Russia; hasanov@pasteurorg.ru (S.A.K.); sasha-khrupina@mail.ru (A.S.V.); annadolgova@inbox.ru (A.S.D.); lavrentieva@pasteurorg.ru (I.N.L.); vgdedkov@yandex.ru (V.G.D.); zarubaev@gmail.com (V.V.Z.)
[2] Division of Pediatric Infectious Diseases, Rambam Health Care Campus, Haifa 3109601, Israel; a_sliita@yahoo.com
[3] Institute of Cytology, Russian Academy of Sciences, 194064 Saint Petersburg, Russia; dkriger@incras.ru
[4] G-INCPM, the Weizmann Institute of Science, Rehovot 7610001, Israel; dmitri.tentler@weizmann.ac.il
[5] N. N. Vorozhtsov Novosibirsk Institute of Organic Chemistry, 630090 Novosibirsk, Russia; ooo@nioch.nsc.ru (O.I.Y.); asokolova@nioch.nsc.ru (A.S.S.), anvar@nioch.nsc.ru (N.F.S.)
[6] A. A. Smorodintsev Influenza Research Institute, 197376 Saint Petersburg, Russia; gorshkov@influenza.spb.ru
[7] Martsinovsky Institute of Medical Parasitology, Tropical and Vector Borne Diseases, Sechenov First Moscow State Medical University, 119435 Moscow, Russia
* Correspondence: esaulkova@pasteurorg.ru; Tel.: +7981-953-53-52

Figure S1: Evaluation of CPE in cells infected with VSV-G and spike-pseudotyped lentiviruses

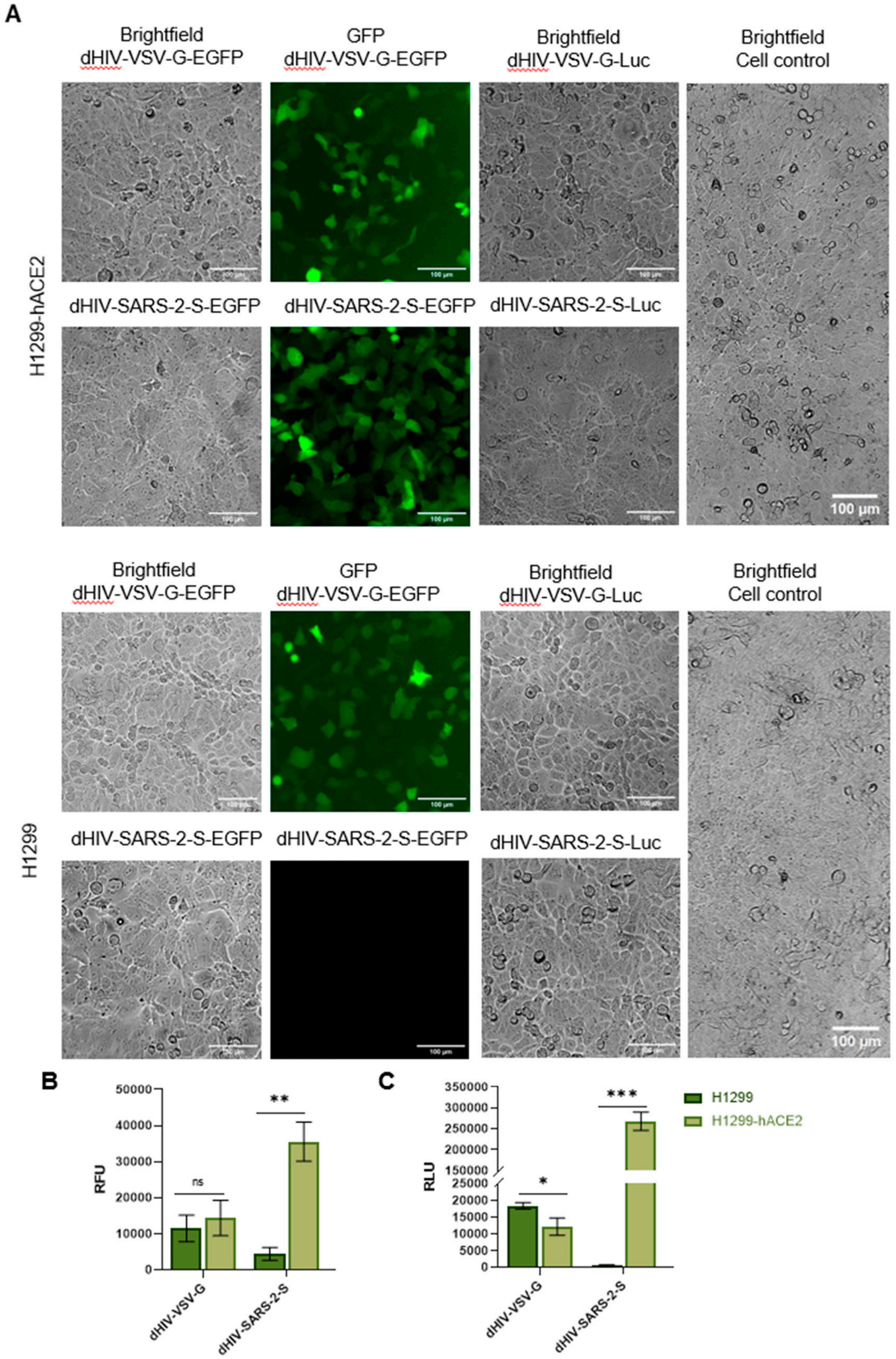

Figure S1. (A) Transduction of H1299 and H1299-hACE2 cell lines by VSV-G and spike-pseudotyped lentiviruses. The results showed that CPE was not observed in both infected cells. (B) Measurement of

luciferase activity in H1299 and H1299-hACE2 cells (n=6) transfected with the dHIV-VSV-G-Luc and dHIV-SARS-2-S-Luc pseudoviruses. (C) Fluorescent analysis of H1299 and H1299-hACE2 cells (n=6) transfected with the dHIV-VSV-G-EGFP and dHIV-SARS-2-S-EGFP pseudoviruses. Statistical significance: $p < 0.05$ (*), $p < 0.01$(**), $p < 0.001$ (***); ns, not significant.

Table S1 and S2: raw data for gene expression analysis for ACE2 and GAPDH

Target	Sample	Control	Expression	Expression SEM	Mean Cq	Cq SEM
ACE2	1 H1299 Control	C	1,00000	0,12874	29,85	0,14980
ACE2	2 H1299 ACE2		1773,26151	77,12438	19,75	0,01577
GAPDH	1 H1299 Control	C			16,45	0,10981
GAPDH	2 H1299 ACE2				17,14	0,06073

Well	Fluor	Target	Content	Sample	Cq
A01	SYBR	GAPDH	Unkn-1	1 H1299 Control	16,30
A02	SYBR	GAPDH	Unkn-1	1 H1299 Control	16,39
A03	SYBR	GAPDH	Unkn-1	1 H1299 Control	16,67
A04	SYBR	ACE2	Unkn-4	1 H1299 Control	30,09
A05	SYBR	ACE2	Unkn-4	1 H1299 Control	29,58
A06	SYBR	ACE2	Unkn-4	1 H1299 Control	29,89
A07	SYBR	GAPDH	NTC		NA
B01	SYBR	GAPDH	Unkn-2	2 H1299 ACE2	17,04
B02	SYBR	GAPDH	Unkn-2	2 H1299 ACE2	17,14
B03	SYBR	GAPDH	Unkn-2	2 H1299 ACE2	17,25
B04	SYBR	ACE2	Unkn-5	2 H1299 ACE2	19,72
B05	SYBR	ACE2	Unkn-5	2 H1299 ACE2	19,78
B06	SYBR	ACE2	Unkn-5	2 H1299 ACE2	19,76
B07	SYBR	ACE2	NTC		NA

Table S3: raw data for fluorescence activity measurement for pseudoviruses dHIV-VSV-G, dHIV-SARS-2-S

Cells	Fluorescence level, RFU			
	H1299		H1299-hACE2	
Pseudovirus	dHIV-VSV-G	dHIV-SARS-2-S	dHIV-VSV-G	dHIV-SARS-2-S
measurement 1	10310	1033	6356	46774
measurement 2	7987	940	5698	52704
measurement 3	9145	1359	7525	48132
measurement 4	7086	1328	7349	55744
measurement 5	7618	1194	7468	54632
measurement 6	9425	979	6415	61295
Blank	1065	789	805	681

Table S4: raw data for luciferase activity measurement for pseudoviruses dHIV-VSV-G, dHIV-SARS-2-S

	Luminescence level, RLU			
Cells	H1299		H1299-hACE2	
Pseudovirus	dHIV-VSV-G	dHIV-SARS-2-S	dHIV-VSV-G	dHIV-SARS-2-S
measurement 1	26030	1295	20320	365300
measurement 2	29920	626,9	19390	391200
measurement 3	23820	402,3	20830	312000
measurement 4	27020	528,4	13570	362600
measurement 5	30740	626,9	16450	347300
measurement 6	31070	547,0	15580	339500
Blank	72,3	121,5	1202	1680

Table S5 and S6: raw data for compounds' cytotoxicity assessment

Concen-tration, µg/ml	Optical density per compound										
	1	2	3	4	5	6	7	8	9	10	Camphe-cene
30	0,2142	0,9076	1,3443	1,1940	1,4135	2,1228	0,1588	1,1095	0,2056	0,1547	1,8552
	0,2023	0,7336	1,8371	1,4340	1,9691	1,9696	0,2379	1,0783	0,2225	0,2444	1,6036
10	2,1891	1,9012	1,6196	1,1489	0,8862	2,4226	0,1128	2,0961	1,5734	1,3422	1,4409
	1,4687	2,2030	1,5207	1,1827	1,2164	2,4462	0,1834	2,0264	1,7843	1,5980	1,1278
3,33	2,6566	2,2060	1,5239	1,2950	0,9761	2,1830	1,7923	1,8842	2,1953	1,8913	1,4530
	2,7468	2,3178	1,5084	1,2373	1,2506	2,4772	2,1347	1,6802	1,8981	2,0331	1,1109
1,11	2,3495	2,3508	1,4562	1,5146	1,2248	2,3710	2,1636	2,2708	2,4134	2,2959	1,3466
	2,1521	2,4245	1,5533	1,5286	1,5302	2,5011	2,4458	1,9128	2,0915	2,2871	1,1370
0,37	2,4503	1,8182	1,5442	1,1372	0,9212	2,3914	2,1470	2,5359	2,3109	2,3710	1,2405
	1,8004	2,3241	1,4274	0,9449	1,2117	1,8205	2,4985	2,1436	2,2839	2,5237	0,9725
0,12	2,2144	2,4483	1,7530	1,0987	1,0443	2,4215	2,2264	2,1854	1,9158	2,0404	1,2202
	2,4876	2,3632	1,4147	1,1025	1,3275	2,2716	2,3845	2,0874	2,1317	2,1556	1,1432
0	2,4359	2,7211	1,5297	1,2082	1,2151	2,1451	1,9488	2,6634	1,9549		1,1147
	2,0587	2,5836	1,7667	1,3368	1,3088	2,4688	2,4152	1,9872	2,1496		1,1497

Concentration, µg/ml	Compound
	umifenovir
300	0,3274
	0,3488
100	0,2193
	0,2364
33,33	0,2301
	0,2913
11,11	2,1281
	2,0862
3,70	2,3938
	2,3186
1,23	2,5614
	2,5369
0,41	2,4440

	2,5864
0,14	2,2505
	2,3243
0,05	2,1901
	2,1483
0,02	2,2090
	2,2854
0	2,3299
	2,2677

Table S7.1 – S7.24: raw data for compounds' anti-pseudovirus activity assessment

7.1 Compound 1 vs LV-Spike RLU

Concentration		Replicates					
definition	µg/ml	1	2	3	4	5	6
CC50/2	5,5	287600	313600	183800	238600	245100	199300
CC50/6	1,8333	393900	405600	285800	364900	364900	327600
CC50/18	0,6111	444000	393400	375600	418000	388300	368600
CC50/54	0,2037	349400	373300	292700	298200	325700	339200
Positive control	0	581800	660800	574000	647300	640800	583900
Negative control	0	3893	4999	5296	5481	5509	5407

7.2 Compound 1 vs LV-G RLU

Concentration		Replicates					
definition	µg/ml	1	2	3	4	5	6
CC50/2	5,5	212300	216500	194300	195200	187500	184100
CC50/6	1,8333	199700	179600	185300	178600	153500	138600
CC50/18	0,6111	164800	161600	157100	158200	130900	163300
CC50/54	0,2037	148200	159200	161600	159400	169300	185300
Positive control	0	167900	167700	169300	168300	155400	137400
Negative control	0	1406	1003	570,4	601,7	451,6	476

7.3 Compound 2 vs LV-Spike RLU

Concentration		Replicates					
definition	µg/ml	1	2	3	4	5	6
CC50/2	11	296100	304700	265900	293500	331800	248800
CC50/6	3,6667	543200	520600	539900	476500	477400	460100
CC50/18	1,2222	545000	425900	512300	559100	392000	510400
CC50/54	0,4074	508700	401100	394300	420100	389300	334200
Positive control	0	638100	463800	476200	569200	451600	444000

Negative control	0	5265	5192	4890	4888	4069	3720

7.4 Compound 2 vs LV-G RLU

Concentration		Replicates					
definition	µg/ml	1	2	3	4	5	6
CC50/2	11	145500	121200	111300	145200	125100	125900
CC50/6	3,6667	146500	154100	131100	155700	158700	122300
CC50/18	1,2222	133800	146600	143400	133500	156600	152100
CC50/54	0,4074	151600	162500	183500	172600	181800	186800
Positive control	0	165200	145900	139500	148600	134200	129600
Negative control	0	1815	1223	1450	1267	1518	1107

7.5 Compound 3 vs LV-Spike RLU

Concentration		Replicates					
definition	µg/ml	1	2	3	4	5	6
CC50/2	15	637700	594700	565800	617000	506400	511800
CC50/6	5	537900	579400	542100	471800	505700	537400
CC50/18	1,6667	394500	445600	421000	452900	458200	426500
CC50/54	0,5556	444600	375500	346300	427800	350900	339600
Positive control	0	612600	639000	723500	507800	611600	
Negative control	0	4135	1557	1318	1270	1124	1014

7.6 Compound 3 vs LV-G RLU

Concentration		Replicates					
definition	µg/ml	1	2	3	4	5	6
CC50/2	15	184000	173000	184700	205300	176400	147000
CC50/6	5	179900	182600	188700	187200	189200	177600
CC50/18	1,6667	152800	168900	168300	153800	140500	147000
CC50/54	0,5556	182000	174200	196600	166400	188200	193400
Positive control	0	148600	134200	129600	168300	155400	137400
Negative control	0	1267	1518	1107	1406	1003	570,4

7.7 Compound 4 vs LV-Spike RLU

Concentration		Replicates					
definition	µg/ml	1	2	3	4	5	6
CC50/2	15	521300	417500	534000	529500	394800	472200
CC50/6	5	520000	521700	538600	486700	489300	516100

CC50/18	1,6667	487300	548300	399000	429800	511600	529100
CC50/54	0,5556	487000	461400	519700	552600	402900	456900
Positive control	0	679100	547900	670600	581200	588800	
Negative control	0	7100	4115	4063	4236	3690	3690

7.8 Compound 4 vs LV-G RLU

Concentration		Replicates					
definition	µg/ml	1	2	3	4	5	6
CC50/2	15	113600	139200	123700	96290	110400	116600
CC50/6	5	301400	281900	274400	286500	314600	282300
CC50/18	1,6667	244300	229000	229400	282900	252000	245200
CC50/54	0,5556	205300	219400	241700	203400	223000	265300
Positive control	0	402300	422900	360800	248100	282100	248500
Negative control	0	2055	1094	1234	782,3	783	570,8

7.9 Compound 5 vs LV-Spike RLU

Concentration		Replicates					
definition	µg/ml	1	2	3	4	5	6
CC50/2	15	311300	409700	284600	295900	417800	228800
CC50/6	5	338500	278300	311400	164400	253700	267200
CC50/18	1,6667	410600	364300	421700	317600	283700	356800
CC50/54	0,5556	594400	397200	180600	380300	212700	148200
Positive control	0	450400	545400	449300	512800	446100	459000
Negative control	0	3744	4440	4270	4503	4214	4358

7.10 Compound 5 vs LV-G RLU

Concentration		Replicates					
definition	µg/ml	1	2	3	4	5	6
CC50/2	15	301300	363300	283100	329700	310300	297100
CC50/6	5	238000	211800	198900	214100	230200	214000
CC50/18	1,6667	189900	179000	182800	204400	167600	172800
CC50/54	0,5556	184600	169500	208300	144600	182600	238200
Positive control	0	319400	337900	311100	260200	246700	201000
Negative control	0	3261	1913	2024	1249	1296	1554

7.11 Compound 6 vs LV-Spike RLU

Concentration		Replicates					
definition	µg/ml	1	2	3	4	5	6
CC50/2	15	352700	481700	367600	345900	442000	331800
CC50/6	5	422900	394800	417700	401700	362900	415900
CC50/18	1,6667	501400	405500	438600	446100	428800	426600
CC50/54	0,5556	655100	482900	275000	700100	461200	264800
Positive control	0	480400	380300	462500	733400	464600	299900
Negative control	0	4618	4347	4306	5185	4705	2573

7.12 Compound 6 vs LV-G RLU

Concentration		Replicates					
definition	µg/ml	1	2	3	4	5	6
CC50/2	15	310500	305600	310400	282000	308200	265000
CC50/6	5	264100	225000	193500	240200	213400	186400
CC50/18	1,6667	217200	189200	170500	181200	185000	181400
CC50/54	0,5556	202600	197800	194800	169000	195300	215400
Positive control	0	402300	422900	360800	260200	246700	201000
Negative control	0	1249	1296	1554	2055	1094	1234

7.13 Compound 7 vs LV-Spike RLU

Concentration		Replicates					
definition	µg/ml	1	2	3	4	5	6
CC50/2	4	456800	552200	481400	414000	489800	466800
CC50/6	1,3333	529200	429900	408600	464800	487900	423200
CC50/18	0,4444	484300	402400	476700	492400	449100	530300
CC50/54	0,1481	702800	462400	289500	648900	478400	282600
Positive control	0	450400	545400	449300	512800	446100	459000
Negative control	0	3744	4440	4270	4503	4214	4358

7.14 Compound 7 vs LV-G RLU

Concentration		Replicates					
definition	µg/ml	1	2	3	4	5	6
CC50/2	4	285500	283000	260600	274600	288300	239000
CC50/6	1,3333	258900	218300	223100	246000	229800	187000
CC50/18	0,4444	196300	176500	192100	210400	163300	185600
CC50/54	0,1481	194000	176900	179000	181000	170000	174200
Positive control	0	318600	302500	315600	246200	255900	214800

Negative control	0	2121	1058	831,8	1146	335,4	482,5

7.15 Compound 8 vs LV-Spike RLU

Concentration		Replicates					
definition	µg/ml	1	2	3	4	5	6
CC50/2	13	418000	343000	305700	281100	356100	323300
CC50/6	4,3333	390300	460600	382900	471900	473400	407200
CC50/18	1,4444	418200	419000	316000	409200	332700	277500
CC50/54	0,4815	257700	246500	225800	227800	245400	198900
Positive control	0	373700	533100	502500	418400	383900	450700
Negative control	0	4618	4347	4306	3893	4999	5296

7.16 Compound 8 vs LV-G RLU

Concentration		Replicates					
definition	µg/ml	1	2	3	4	5	6
CC50/2	13	341600	291300	275600	327900	315300	281600
CC50/6	4,3333	282400	247100	240900	249700	235500	211700
CC50/18	1,4444	202100	203500	212000	202000	194900	207800
CC50/54	0,4815	229400	185400	209100	203100	169300	190500
Positive control	0	291300	241100	261300	241600	222400	154300
Negative control	0	2679	1867	1264	1451	1752	1295

7.17 Compound 9 vs LV-Spike RLU

Concentration		Replicates					
definition	µg/ml	1	2	3	4	5	6
CC50/2	7	271900	250100	228400	223500	222200	211700
CC50/6	2,3333	402200	419500	425200	406800	360200	392900
CC50/18	0,7778	424400	416000	440600	415900	425600	396200
CC50/54	0,2593	316200	344500	328100	309200	264600	301900
Positive control	0	469900	451300	500200	462300	400500	443300
Negative control	0	4503	4214	4358	7100	4115	4063

7.18 Compound 9 vs LV-G RLU

Concentration		Replicates					
definition	µg/ml	1	2	3	4	5	6
CC50/2	7	198600	219700	221400	239700	225700	198900
CC50/6	2,3333	262600	239600	200600	251900	214600	223200

CC50/18	0,7778	220700	206700	192200	201800	191600	196700
CC50/54	0,2593	209100	175100	206200	181000	170100	184200
Positive control	0	241600	222400	154300	318600	302500	315600
Negative control	0	1146	335,4	482,5	2679	1867	1264

7.19 Compound 10 vs LV-Spike RLU

Concentration		Replicates					
definition	µg/ml	1	2	3	4	5	6
CC50/2	14	3371	5266	4717	8003	24640	11490
CC50/6	4,6667	296400	334300	365600	360300	326400	414000
CC50/18	1,5556	398400	337200	433500	392800	372800	433100
CC50/54	0,5185	375400	371200	380400	346000	351700	378700
Positive control	0	356200	366900	471200	380600	400300	401300
Negative control	0	735,7	618,1	377,8	309,5	449,4	2573

7.20 Compound 10 vs LV-G RLU

Concentration		Replicates					
definition	µg/ml	1	2	3	4	5	6
CC50/2	14	2213	2529	2423	857,1	1171	1635
CC50/6	4,6667	138800	148200	150000	132100	138100	153100
CC50/18	1,5556	220800	200900	212400	239000	211900	179100
CC50/54	0,5185	171200	159500	227800	187500	192200	226800
Positive control	0	312200	372700	268400	296300	216500	227200
Negative control	0	1303	768,9	641,3	784	576,1	744,4

7.21 Camphecene vs LV-Spike RLU

Concentration		Replicates					
definition	µg/ml	1	2	3	4	5	6
	30	72330	105300	91650	58110	99690	76160
	10	195300	141800	218200	132500	124300	232400
	3,3333	179800	258100	213800	161800	248900	204400
	1,1111	209000	250700	278000	262200	215700	241200
Positive control	0	265500	304600	260600	344500	278000	399000
Negative control	0	1830	735,7	618,1	377,8	309,5	449,4

7.22 Camphecene vs LV-G RLU

Concentration		Replicates					
definition	µg/ml	1	2	3	4	5	6
	30	163700	178800	158400	159900	227700	179800
	10	179600	158600	140300	150600	147900	136300
	3,3333	132800	121900	130800	137000	125900	127900
	1,1111	114400	119400	116500	123700	112500	148700
Positive control	0	263100	329400	200000	246900	180800	195100
Negative control	0	2566	1837	1710	1782	1294	867,8

7.23 Umifenovir vs LV-Spike RLU

Concentration		Replicates					
definition	µg/ml	1	2	3	4	5	6
	10	848000	792400	820600	826300	758500	704200
	3,3333	784700	765000	924300	778000	878800	971200
	1,1111	885000	798800	918800	814500	754800	902800
	0,3704	979800	1004000	1076000	918100	1014000	1118000
Positive control	0	812200	874300	962500	875000	936900	1072000
Negative control	0	6552	3838	7520	8898	7567	5474

7.24 Umifenovir vs LV-G RLU

Concentration		Replicates					
definition	µg/ml	1	2	3	4	5	6
	10	109800	134700	101300	93300	117500	114200
	3,3333	214100	156800	158000	176600	156900	170900
	1,1111	157200	152500	172500	152000	140100	130100
	0,3704	168700	182100	154400	165000	160500	160200
Positive control	0	296300	216500	227200	263100	329400	200000
Negative control	0	784	576,1	744,4	2566	1837	1710

Table 8: Luciferase_activity_measurement

	Luminescence level, RLU			
Cells	H1299		H1299-hACE2	
Pseudovirus	dHIV-VSV-G	dHIV-SARS-2-S	dHIV-VSV-G	dHIV-SARS-2-S
measurement 1	17020	285,6	10080	255600
measurement 2	17980	599,8	10490	246300
measurement 3	18140	175	9346	292500
measurement 4	19000	912,1	13630	247500
measurement 5	19880	505,3	15890	272400

measurement 6	18340	455,1	13810	295300
Blank	174,2	117,6	2027	2234

Table 9: Fluorescence_activity_measurement

Cells	Fluorescence level, RFU			
	H1299		H1299-hACE2	
Pseudovirus	dHIV-VSV-G	dHIV-SARS-2-S	dHIV-VSV-G	dHIV-SARS-2-S
measurement 1	15190	10219	13342	31539
measurement 2	17979	10606	16026	44184
measurement 3	14730	9861	20664	36214
measurement 4	19202	12541	17458	44473
measurement 5	23627	14002	21669	42683
measurement 6	22525	13489	27273	44288
Blank	7955	6786	3711	6391
